# A Rapid Review of Adenocarcinoma and Pulmonary Tumor Thrombotic Microangiopathy: A Deadly Duo

**DOI:** 10.7759/cureus.76842

**Published:** 2025-01-03

**Authors:** Emin Gayibov, Amin H Karim

**Affiliations:** 1 Third Faculty of Medicine, Charles University, Prague, CZE; 2 Department of Cardiovascular Disease, Baylor College of Medicine, Houston, USA; 3 Department of Cardiovascular Disease, Weill Medical College of Cornell University, New York City, USA; 4 Department of Cardiovascular Disease, Methodist Academy of Medicine, Houston, USA

**Keywords:** adenocarcinoma, neoplasms, paraneoplastic syndromes, pulmonary tumor thrombotic microangiopathy, thrombotic microangiopathies

## Abstract

Pulmonary tumor thrombotic microangiopathy (PTTM) is a rare paraneoplastic syndrome associated with various adenocarcinomas, most commonly gastric adenocarcinoma. This condition can progressively worsen pulmonary arterial hypertension, leading to acute or subacute pulmonary heart failure and respiratory insufficiency. This paper examines the pathogenesis, clinical presentation, diagnosis, treatment, and prognosis of PTTM. Given PTTM's poor prognosis, we emphasize treatment strategies. PTTM in adenocarcinoma patients can mimic other pulmonary diseases, causing diagnostic delays. Current PTTM treatment strategies primarily focus on managing the underlying malignancy and addressing thrombotic complications. Anti-angiogenic therapy with bevacizumab and the platelet-derived growth factor receptor antagonist imatinib have shown promise in multiple cases. Further research is needed to develop more effective and targeted therapies for this challenging condition. The precise mechanisms underlying this association remain to be fully elucidated.

## Introduction and background

Paraneoplastic syndromes constitute a diverse group of clinical disorders associated with malignant diseases that can significantly impact patient morbidity and mortality. They are not directly attributable to the physical effects of the primary or metastatic tumor [1]. In these syndromes, malignant cells do not directly cause symptoms related to metastasis; rather, they induce the generation of autoantibodies, cytokines, hormones, or peptides that exert effects on multiple organ systems [2]. It is estimated that paraneoplastic syndromes affect up to 8% of patients with cancer [3]. Table 1 provides a concise overview of commonly encountered paraneoplastic syndromes [4-23].

**Table 1:** A concise overview of paraneoplastic syndromes including pulmonary tumor thrombotic microangiopathy Source: [4-23] SCLC, small cell lung carcinoma; SIADH, syndrome of inappropriate antidiuretic hormone secretion

Organ system	Paraneoplastic syndrome	Most associated cancer type	Clinical signs
Neurological	Paraneoplastic encephalitis	SCLC, testicular cancer	Cognitive dysfunction, seizures, personality changes, hallucinations, autonomic dysfunction
	Subacute cerebellar degeneration	Breast, ovarian, SCLC, Hodgkin lymphoma	Ataxia, dysarthria, dizziness, diplopia, nausea, vomiting
	Opsoclonus-myoclonus syndrome	Neuroblastoma (children), SCLC (adults)	Rapid eye movements, body jerks, ataxia, hypotonia
	Myasthenia gravis	Thymoma	Muscle weakness, ptosis, diplopia, diaphragmatic weakness
	Lambert-Eaton myasthenic syndrome	SCLC	Proximal muscle weakness, autonomic dysfunction, diminished reflexes
	Autonomic neuropathy	SCLC, thymoma	Dry mouth, altered pupillary reflexes, orthostatic hypotension, GI dysfunction
	Subacute sensory neuropathy	SCLC	Paresthesia, neuropathic pain, diminished sensation
Endocrine	Cushing syndrome	SCLC, pancreatic cancer	Hypertension, centripetal obesity, hypokalemia, edema
	SIADH	SCLC	Hyponatremia, lethargy, confusion, seizures
	Hypercalcemia	Lung, renal cell, multiple myeloma	Lethargy, nausea, bradycardia, short QT interval on ECG
	Non-islet tumor hypoglycemia	Fibrosarcoma, hepatocellular carcinoma	Hypoglycemia, confusion, seizures
	Carcinoid syndrome	Bronchial carcinoid, pancreatic carcinoma	Flushing, diarrhea, bronchospasm
	Hyperaldosteronism	Adrenal adenoma, non-Hodgkin lymphoma	Hypertension, hypokalemia
Rheumatological	Paraneoplastic polyarthritis	Various malignancies	Migratory, asymmetric arthritis
	Polymyalgia rheumatica	Myelodysplastic syndrome	Pain and stiffness in shoulders, neck, and hips
	Hypertrophic osteoarthropathy	Lung cancer	Digital clubbing, joint swelling, periostitis
	Multicentric reticulohistiocytosis	Various malignancies	Papules, nodules, destructive polyarthritis
Hematological	Polycythemia	Renal cell carcinoma, cerebellar hemangioma	Increased hemoglobin, pallor, fatigue
	Trousseau syndrome (migratory thrombophlebitis)	Pancreatic, Bronchogenic carcinoma	Painful migratory thrombophlebitis
Dermatological	Acanthosis nigricans	Gastric adenocarcinoma	Hyperpigmented, velvety plaques in axilla, neck
	Paraneoplastic pemphigus	B-cell lymphoproliferative disorders	Blisters, mucosal erosions
	Sweet syndrome	Hematological malignancies	Painful, erythematous plaques with fever
	Dermatomyositis	Ovarian, lung, pancreatic cancer	Heliotrope rash, Gottron papules, proximal muscle weakness
Renal	Nephrotic syndrome	Various tumors	Proteinuria, edema, fluid overload
	Electrolyte imbalances	SCLC, thymoma	Hyponatremia, hyperphosphatemia, acid-base disturbances
Pulmonary	Pulmonary tumor thrombotic microangiopathy	Various adenocarcinomas	Microvascular thrombosis, endothelial injury, and severe pulmonary arterial hypertension
	Lymphangitic carcinomatosis	Various adenocarcinomas	Progressive dyspnea, cough, and hypoxemia
	Pulmonary alveolar proteinosis	Leukemia, lymphoma	Progressive respiratory insufficiency

Pulmonary tumor thrombotic microangiopathy (PTTM) is a rare and often underrecognized paraneoplastic syndrome associated with various adenocarcinomas, often linked to gastric adenocarcinoma [24]. Although less frequently observed, breast, lung, and urothelial malignancies have also been linked to PTTM [25]. Post-mortem examinations of carcinoma patients indicate a PTTM prevalence ranging from 1.4% to 3.3% [24,26]. This condition can lead to a progressive deterioration of pulmonary arterial hypertension (PAH), culminating in acute or subacute pulmonary heart failure and respiratory insufficiency. Hence, early recognition and treatment are crucial to prevent irreversible lung damage and complications. First described by von Herbay et al. in 1990 [26], PTTM is characterized by the presence of numerous tumor emboli within the pulmonary microvasculature, leading to microvascular thrombosis and endothelial injury. Later studies have highlighted the critical role of tumor-derived factors in driving hypercoagulability and angiogenesis, as explained in the pathogenesis section.

In this paper, we will examine the pathogenesis, clinical presentation, diagnosis, treatment, and prognosis of PTTM in patients with adenocarcinoma of various origins. Given the poor prognosis associated with PTTM, we will place particular emphasis on treatment strategies. We will discuss the approaches taken in different cases in recent years to inform current best practices, including their limitations and potential avenues for improvement. This rapid review will delve into the current understanding of this rare paraneoplastic syndrome and its potential clinical implications. By reviewing the available literature, we aim to shed light on the pathophysiology, clinical manifestations, diagnostic challenges, and therapeutic options for PTTM.

## Review

Methodology

All the studies referred for this rapid review have been searched via the National Library of Medicine (NIH), PubMed, with the free-text keywords “pulmonary tumor thrombotic microangiopathy (PTTM)”, “PTTM AND adenocarcinoma”, “pathogenesis AND PTTM”, “clinical presentation AND PTTM”, “diagnosis AND PTTM”, “treatment AND PTTM”, and “prognosis AND PTTM”. A visual tool Connected Papers [27] and reference manager software Mendeley [28] served for proper citation and access to the studies regarding the abovementioned aspects of PTTM. For this rapid review, we did not specify the time range of studies referred due to insufficiency in number. Our inclusion criteria comprised type I tumor embolism, which is the "classic" or "true" type originating from a distant primary tumor via hematologic seeding with no invasion into vessel walls. Among type I tumor embolism studies, we included ones that specifically focused on the PTTM, either antemortem or postmortem, as a confirmed diagnosis. We disregarded the studies involving type II tumor embolism, which results from a tumor growing into the pulmonary arteries with invasion into vessel walls. The search strategy is summarized in Table 2.

**Table 2:** Summary of the search strategy for this rapid review

Items	Specification
Date of search	1 October 2024 to 17 November 2024
Databases and other sources searched	National Library of Medicine, PubMed
Search terms used	“pulmonary tumor thrombotic microangiopathy (PTTM)”, “PTTM AND adenocarcinoma”, “pathogenesis AND PTTM”, “clinical presentation AND PTTM”, “diagnosis AND PTTM”, “treatment AND PTTM”, and “prognosis AND PTTM”
Timeframe	No specific timeframe
Inclusion and exclusion criteria	Case reports and reviews were included if they specifically focused on PTTM as type I tumor embolism, the "classic" or "true" type originating from a distant primary tumor via hematologic seeding without vessel wall invasion. Studies involving type II tumor embolism, resulting from a tumor growing into the pulmonary arteries with vessel wall invasion, and mixed embolism were excluded
Selection process	A total of 164 studies were initially identified, of which 55 were ultimately included Author E.G. conducted the literature selection. A.K. supervised the search strategy.
Any additional considerations	Connected Papers [27] and Mendeley [28] served for proper citation and access to the studies

Pathogenesis of pulmonary tumor thrombotic microangiopathy

Associated with mucin-secreting adenocarcinomas, PTTM is a specific type of pulmonary tumor embolism. It is classified within type I tumor embolism, the "classic" or "true" type originating from a distant primary tumor via hematologic seeding without vessel wall invasion [29,30]. In contrast, type II tumor embolism arises from a tumor growing into the pulmonary arteries with invasion into vessel walls [31]. In terms of histopathology, PTTM is characterized by fibrocellular intimal proliferation of small and medium pulmonary arteries and arterioles with the presence of tumor emboli [26,32]. This distinctive feature can be observed using various histological staining techniques, such as Verhoeff-Van Gieson and alpha-smooth muscle actin immunohistochemistry [33,34]. Fibrocellular intimal proliferation involves the growth of cells, primarily smooth muscle cells and fibroblasts, and extracellular matrix within the intima of small pulmonary arteries and arterioles. This leads to narrowing of the vessel lumen. Reactive fibrointimal thickening and occlusion were also revealed in the pulmonary lymphatic vessels and veins in a particular PTTM case [35]. It has also been reported that PTTM is frequently associated with lymphangiosis carcinomatosa, a serious condition that occurs when cancer cells spread to the lymph vessels, causing inflammation and blockage and becoming a predisposing factor to PTTM [26,36]. In PTTM, multiple microscopic tumor cells become stuck to the inner lining of small pulmonary blood vessels. This triggers an inflammatory response, causing the vessel walls to thicken and narrow. Additionally, blood clots form within these vessels, further restricting blood flow. As a result, the lung's blood vessel network becomes severely compromised, leading to PAH [37,38].

In PTTM, tumor cells not only physically obstruct blood vessels but also release tissue factor (TF), which activates the blood clotting system. They also release inflammatory mediators that promote inflammation and growth factors that stimulate the growth of vessel tissue. This excessive growth can lead to a thickening of the vessel walls, narrowing the blood passage and further hindering the blood flow. Platelet-derived growth factor (PDGF) and vascular endothelial growth factor (VEGF) produced by tumor cells, and versican (VCAN), an extracellular matrix proteoglycan, are considered to be the important factors involved in pulmonary vascular remodeling seen in PTTM [39-41]. Growth factors, particularly transforming growth factor-β (TGF-β), stimulate the expression of VCAN. VCAN binds to growth factors and other extracellular matrix components, creating a local reservoir for these factors [42]. Furthermore, VCAN-hyaluronan aggregates occur in various human lung diseases, including PAH, and provide a permissive environment for arterial smooth muscle growth [41]. These aggregates can be visualized using specific staining techniques with primary antibodies targeting VCAN and hyaluronan-binding protein [43]. Tumor necrosis factor-α, another cytokine released by tumor cells, induces changes in endothelial cell functions, such as upregulation of TF, contributing to the activation of blood clotting system [44].

As previously stated, PTTM is strongly associated with mucin-secreting adenocarcinomas. Mucin production by these tumors likely contributes to the development of PTTM through several mechanisms. Mucinous material can encapsulate tumor cells, forming emboli that travel to the lungs and cause vascular obstruction. Additionally, mucin can stimulate inflammation and damage the vessel walls, further promoting thrombosis. The pro-inflammatory nature of mucin can activate coagulation pathways, leading to thrombus formation [45,46]. While the exact mechanisms are still under investigation, it is clear that mucin plays a significant role in the pathogenesis of this complex disease. Furthermore, circulating tumor-derived, TF-positive microparticles are strongly associated with the activation of blood coagulation, another potential mechanism contributing to the pathogenesis of PTTM that warrants further investigation [47]. Tumor cells express TF and spontaneously release TF-positive microparticles into the bloodstream. Microparticles are small membrane vesicles that exhibit high procoagulant activity. It has been proposed that these circulating tumor-derived, TF-positive microparticles may contribute to the increased rates of thrombosis, including PTTM, as observed in cancer patients [47]. Further contribution to the prothrombotic state can be via a decrease in ADAMTS13 activity. ADAMTS13 is an enzyme responsible for cleaving von Willebrand factor (VWF), a protein involved in blood clotting [48]. Reduced ADAMTS13 leads to an accumulation of large, multimeric forms of VWF. These large VWF multimers are more potent at binding platelets and promoting their aggregation [48,49]. This increased platelet aggregation significantly increases the risk of blood clot formation, contributing to the development of venous thromboembolism and other thrombotic complications in cancer patients, possibly including PTTM.

**Figure 1:**
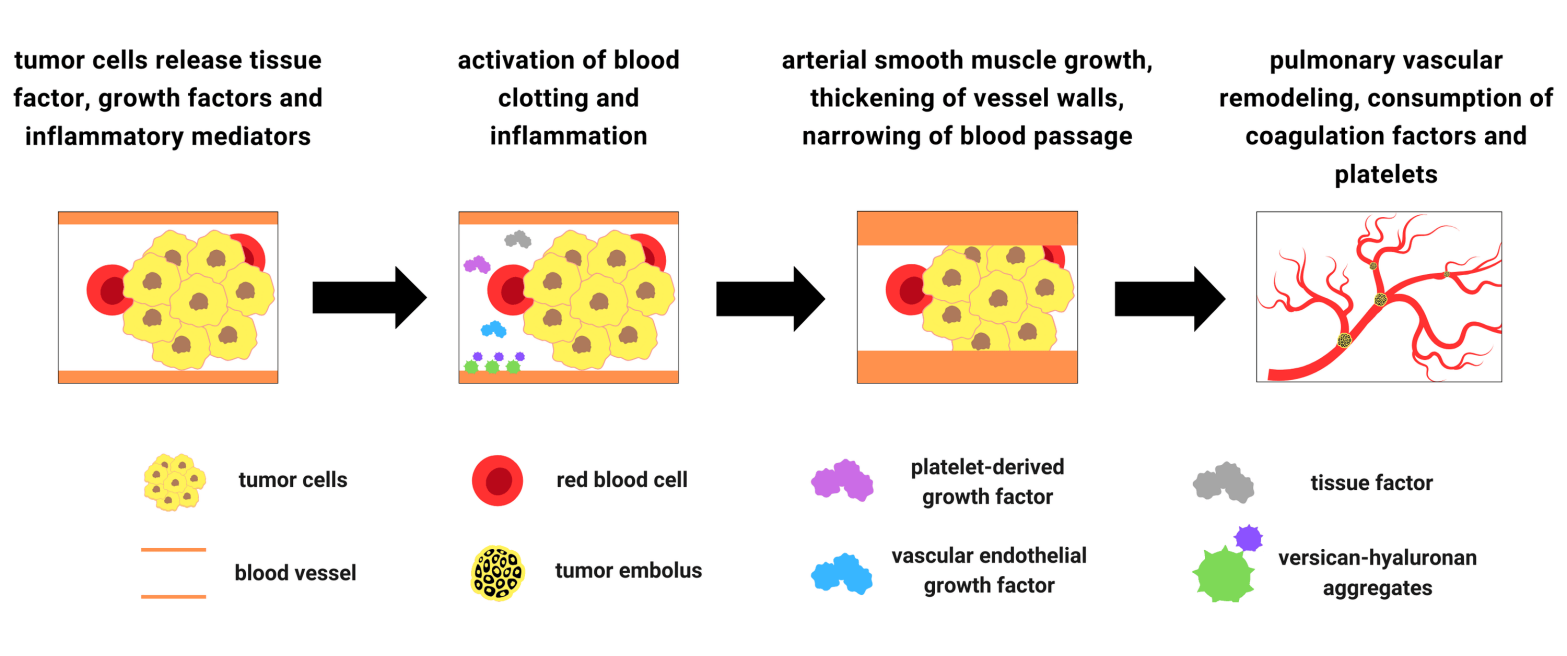
Summary of the pathogenesis of pulmonary tumor thrombotic microangiopathy

The interaction between the tumor emboli and endothelial cells via multiple mediators eventually results in the consumption of coagulation factors and platelets, consistent with commonly found laboratory findings, including thrombocytopenia, and disseminated intravascular coagulation (DIC) [25]. Subsequent impaired fibrinolysis in PTTM disrupts the body's ability to dissolve blood clots in the lungs. This occurs due to an imbalance between clot formation and breakdown, with factors such as increased levels of plasminogen activator inhibitor-1 and decreased levels of tissue plasminogen activator playing significant roles [50]. The resulting persistent blood clots contribute to the development of PAH, right ventricular failure, and other serious complications. The pathogenesis of PTTM is summarized in Figure 1. While growth factors such as PDGF, VEGF, and TGF-β are implicated, the precise molecular pathways driving tumor cell proliferation and invasion in PTTM remain unclear. The mechanisms by which tumor cells arrest in the pulmonary circulation and the role of specific inflammatory mediators in this process need further investigation. The complex interplay of these factors highlights the intricate mechanisms underlying PTTM and emphasizes the need for further research to unravel the precise molecular pathways involved.

Clinical presentation and diagnosis of pulmonary tumor thrombotic microangiopathy

Multiple case reports show that the most common symptoms of PTTM include cough, sputum production, shortness of breath, inability to lie down at night, chest tightness, chest pain, hemoptysis, fever, malaise, and wasting. Additionally, fatigue, weight loss, fluid buildup in the legs and ankles, and bluish discoloration of the skin are also reported systemic symptoms [51-54]. In clinical settings, PTTM is primarily a preliminary diagnosis made through a combination of the patient's medical history, physical examination, echocardiographic and CT imaging, and laboratory tests in patients with cancer. The recognition of PTTM in cancer patients is crucial for several reasons. Firstly, PTTM can significantly worsen the prognosis of cancer patients by accelerating disease progression and increasing the risk of thromboembolic events. Secondly, early diagnosis and appropriate management of PTTM can help alleviate respiratory symptoms and improve quality of life. A diagnosis of PTTM requires a high index of suspicion, especially in patients with sudden onset dyspnea but no radiological findings pointing to pulmonary embolism [55]. Clinicians should be vigilant for PTTM in patients with cancers such as breast, lung, and ovarian, especially those undergoing chemotherapy or with advanced disease. Key symptoms include sudden shortness of breath, chest pain, cough, and hemoptysis [51-54]. Thorough evaluation, including blood tests, chest imaging, and consideration of other diagnoses such as pulmonary embolism and pneumonia, is essential. The challenge in the diagnosis of PTTM can be explained by the low rate of antemortem versus a high rate of postmortem diagnosis, as nearly 80% of identified cases were diagnosed by autopsy [25]. A study by Bak et al. highlights the challenge of diagnosing PTTM. Over 10 years at a tertiary center, they collected 28 cases suspected of PTTM, with only one confirmed histologically [56]. This underrecognition can be further attributed to its nonspecific clinical presentation, rapid disease progression, and the overall rarity of the condition, which often delays consideration in the differential diagnosis.

The clinical presentation of PAH, right ventricular failure, and abnormal laboratory studies including elevation in D-dimer and prothrombin time, the presence of anemia and thrombocytopenia, and DIC should prompt consideration of PTTM [25,57]. Schistocytes may be present in the peripheral blood smear in PTTM, as red blood cells can be sheared by the thickened intima. However, their presence alone is not diagnostic and should be interpreted in the context of other clinical and laboratory findings, such as those associated with thrombotic thrombocytopenic purpura [38]. The elevated D-dimer likely reflects the increased fibrin turnover associated with microthrombi formation in PTTM. Similarly, a prolonged prothrombin time can indicate a consumption coagulopathy, a hallmark of PTTM. Anemia and thrombocytopenia are likely secondary to microvascular thrombosis and platelet consumption in the setting of DIC. The presence of these laboratory abnormalities together with respiratory deterioration suggests PTTM [25,57].

PTTM should also be considered in rapidly dyspneic cancer patients with the presence of PAH on echocardiography without any apparent cause, and with the absence of pulmonary arterial thrombus on chest computed tomography (CT) [57]. Use of pulmonary aspiration cytopathology for antemortem diagnosis has been reported in a few cases [51,58]. For imaging, the use of 18F-fluorodeoxyglucose positron emission tomography/CT has been reported in multiple studies [24,59,60]. Case reports by Tashima et al. [59] and Kamada et al. [60] demonstrated multiple foci with abnormal FDG uptake in both lungs affected by PTTM.

Chronic pulmonary thromboembolism (CTEPH) is a distinct clinical entity that can be challenging to differentiate from PTTM. While both conditions can present with respiratory symptoms, their clinical manifestations and diagnostic approaches differ. In contrast to the acute presentation of PTTM, CTEPH often has a more insidious onset with less prominent respiratory symptoms. Cough, a common feature of acute PTTM, is less frequently observed in CTEPH. CT findings can also be helpful in differentiating the two conditions. In PTTM cases, ground-glass opacities, nodules, mediastinal and hilar lymphadenopathy, and septal thickening are often revealed. In contrast, CTEPH cases may demonstrate mosaicism, wedge-shaped infarcts, organized and calcified thrombus, and enlarged bronchial artery [57]. Additionally, laboratory findings in CTEPH are typically nonspecific and may not reveal the characteristic thrombocytopenia and DIC seen in PTTM [25,61]. Table 3 summarizes the clinical presentation and diagnosis of PTTM.

**Table 3:** Summary of the clinical presentation and diagnosis of pulmonary tumor thrombotic microangiopathy Source: [24,25,38,51,57-60] CT, computed tomography; FDG-PET, 18F-fluorodeoxyglucose positron emission tomography; PAH, pulmonary arterial hypertension

Feature	Description
Common symptoms	Cough, sputum production, shortness of breath, inability to lie flat, chest tightness, chest pain, hemoptysis, fever, malaise, and wasting
Diagnostic approach	Combination of medical history, physical examination, imaging (echocardiography, CT), and laboratory tests
Importance of early diagnosis	A delay in diagnosis worsens prognosis, increases risk of thromboembolic events, and negatively impacts quality of life
Diagnostic challenges	High rate of postmortem diagnosis, difficulty in differentiating from other pulmonary conditions, and PAH on echocardiography and CT without any apparent cause
Clinical presentation	PAH, right ventricular failure, abnormal laboratory findings (increase in D-dimer and prothrombin time, anemia, thrombocytopenia, DIC), and schistocytes in peripheral blood smear
Diagnostic tools	Pulmonary aspiration cytopathology, FDG-PET/CT. Chest CT may not show typical pulmonary embolism. FDG-PET/CT can reveal multiple lung nodules

Treatment and prognosis of pulmonary tumor thrombotic microangiopathy

The choice of medication depends on the individual patient and the severity of their condition. Apart from oxygen therapy, the medications used to treat PTTM include those for advanced PAH, such as sildenafil, tadalafil, ambrisentan, bosentan, and epoprostenol [62]. Diuretics such as furosemide and spironolactone help reduce fluid overload, while corticosteroids such as dexamethasone and prednisone have anti-inflammatory effects. Given that PTTM presents with consumptive coagulopathy, anticoagulants should be used with caution. In such cases, low-molecular-weight heparin may be the optimal strategy [63]. In pulmonary embolism, the pathology is primarily caused by the activation of the blood coagulation cascade, but in PTTM, fibroproliferative changes in the vascular lumen arising from TF release are the prominent causes. Therefore, while anticoagulant therapy may be effective for pulmonary embolism, it cannot improve PTTM. In the case of right heart failure, intravenous diuresis, inotropic support, and pulmonary vasodilator therapy should be considered [54]. A multidisciplinary approach involving pulmonologists, oncologists, and other specialists is essential for the optimal management of PTTM patients. A patient-centered approach, which includes timely supportive care and symptom management, can improve quality of life and alleviate symptoms.

At present, PTTM management lacks a consistent, evidence-based approach due to the condition's rarity and rapid progression, which frequently leads to late diagnosis and intervention. Almost all patients with PTTM die within a week of the dyspnea onset due to progressive PAH, subacute right heart failure, or sudden death [53,64]. The rarity of PTTM poses significant challenges to research, including small sample sizes and difficulty in recruitment for clinical trials. While anti-inflammatory therapy with corticosteroids has been frequently utilized as a potential treatment strategy for PTTM, its efficacy remains uncertain [53,65-67]. A case report by Miyazaki et al. demonstrated a temporary improvement in lung function and right ventricular pressure overload following corticosteroid administration [53]. However, the patient ultimately succumbed to respiratory failure, highlighting the limitations of current therapeutic approaches and the need for further research to identify effective interventions for this challenging condition. Other studies similarly reported the ineffectiveness of anti-coagulants and corticosteroids [65-67].

Clinical reports suggest that bevacizumab may be a valuable therapeutic option for patients with PTTM, especially when used in conjunction with other therapies. Higo et al. presented a case study of a colorectal cancer patient exhibiting PTTM who underwent a combination therapy involving imatinib, a PDGF receptor antagonist, bevacizumab, a VEGF receptor inhibitor, and the chemotherapeutic agents S-1 and cisplatin [68]. Following this treatment regimen, the patient exhibited a significant improvement in symptoms without experiencing a deterioration of PAH. However, 12 months post-treatment, the patient succumbed to respiratory failure secondary to an influenza infection. Despite this outcome, the authors posit that the molecular-targeted drugs employed in the therapy were efficacious in managing PTTM based on the patient's clinical trajectory [68]. Kotake et al. reported a significant improvement of PTTM with lung adenocarcinoma in terms of PAH, respiratory symptoms, and other outcomes after bevacizumab treatment combined with paclitaxel and carboplatin [69]. Taniguchi et al. reported a similar improvement in uterine cancer-induced PTTM after successful treatment with platinum-based chemotherapy and bevacizumab [70]. These case studies suggest that bevacizumab, in combination with other therapies, may be a promising treatment option for PTTM, especially when used in conjunction with targeted therapies. Bevacizumab therapy can be associated with adverse effects. The most common side effect is hypertension, which requires regular blood pressure monitoring and effective management with antihypertensive medications. Proteinuria, thromboembolism, impaired wound healing, and bleeding are other clinically encountered side effects [71]. In more severe cases, a minority of patients may develop thrombotic microangiopathy (TMA). Bevacizumab-associated TMA, along with other drug-induced TMAs, is currently an indication for drug discontinuation due to poor prognosis, including acute kidney injury often requiring dialysis and progression to chronic kidney disease. Most cases, however, improve after discontinuation of bevacizumab [72].

Imatinib has demonstrated promising results in addressing complications associated with PTTM, as evidenced by several case studies. The efficacy of imatinib in treating a patient with PAH associated with PTTM has been demonstrated by the case study of Ogawa et al. [73]. Following imatinib therapy, the patient experienced a dramatic reduction in PAH, enabling successful weaning from percutaneous cardiopulmonary support within a 20-day timeframe. Based on these findings, the authors suggested that imatinib may be a viable therapeutic option for alleviating PAH arising from PTTM [73]. Kimura et al. [74] reported a case of a breast cancer patient with PTTM who experienced a dramatic improvement with bevacizumab therapy. The patient received paclitaxel and bevacizumab for one year, successfully controlling the condition and extending their survival to one year and eight months. This case highlighted the potential efficacy of bevacizumab in managing PTTM associated with breast cancer [74]. Another case study reported that bevacizumab combined with pemetrexed significantly improved lung adenocarcinoma-induced PTTM respiratory dysfunction [52]. Yoshikawa et al. also reported a case of PTTM associated with metastatic breast cancer, which exhibited a significant improvement of respiratory dysfunction and PAH after imatinib was administered [75]. Similarly, imatinib dramatically alleviated the PTTM induced by gastric cancer in another case report by Kubota et al [76]. These findings underscore the potential of imatinib as a targeted therapy for PTTM. Yet still, imatinib can cause a range of cutaneous side effects, along with fever and diarrhea. A maculopapular rash is the most common. For many patients experiencing intolerable side effects, temporarily reducing the dose can help resolve the issue [77]. Furthermore, while both bevacizumab and imatinib are approved medications, access can be challenging due to factors such as cost, insurance coverage, and availability. Similar to bevacizumab, imatinib can also result in drug-mediated TMA, as reported in two cases [78]. Therefore, the administration of bevacizumab and imatinib should be considered carefully, taking into account the potential for drug-mediated TMA. Table 4 summarizes the referred studies regarding the treatment and prognosis of adenocarcinoma-induced PTTM.

**Table 4:** Summary of referred studies regarding the treatment and prognosis of PTTM DIC, disseminated intravascular coagulation; PAH, pulmonary arterial hypertension; PTTM, pulmonary tumor thrombotic microangiopathy

Study author(s)	Type of study	Patient cancer type	Treatment	Outcome
Lu et al. [52]	Case report	Lung adenocarcinoma	Bevacizumab, pemetrexed	Successful improvement in chest CT findings, respiratory symptoms, DIC. On the second day, dyspnea improved, and The patient could complete walking exercises. Cough was gradually relieved, without any further hemoptysis, together with significantly improved fatigue, sleep, food intake, and mental and physical status
Miyazaki et al. [53]	Case report	Gastric cancer	Corticosteroids	Temporary improvement in lung function and right ventricular pressure, but eventual death from respiratory failure
Higo et al. [68]	Case report	Colorectal cancer	Imatinib, bevacizumab, s-1, cisplatin	Significant improvement in symptoms without deterioration of PAH, but eventual death from respiratory failure
Kotake et al. [69]	Case report	Lung adenocarcinoma	Carboplatin, paclitaxel, and bevacizumab	Successful improvement in PAH, respiratory symptoms, and other outcomes. On day 10, oxygen saturation rate was improved to 95%, and she was discharged after recovery was confirmed
Taniguchi et al. [70]	Case report	Uterine cancer	Carboplatin, paclitaxel, and bevacizumab	Patient's respiratory status and radiological findings improved concomitantly with a reduction in the size of the tumor. The patient recovered well from respiratory failure and her condition has improved, even six months after the end of treatment
Ogawa et al. [73]	Case report	Gastric and duodenal carcinoma	Imatinib	Dramatic amelioration of a PAH patient was able to be weaned from percutaneous cardiopulmonary support within 20 days of treatment
Kimura et al. [74]	Case report	Stage IV left-sided breast cancer	Paclitaxel and bevacizumab for breast cancer and concurrent treatment for PAH and DIC	Successful control of the condition with paclitaxel and bevacizumab for a year. The patient survived for 1 year and 8 months
Yoshikawa et al. [75]	Case report	Metastatic breast cancer	Imatinib	Patient exhibited significant improvement of respiratory dysfunction and PAH
Kubota et al. [76]	Case report	Gastric cancer, signet-ring cell carcinoma	Imatinib	Significant decrease in mean pulmonary arterial pressure five days after imatinib administration. The patient was discharged and lived without symptoms of PAH until her death due to systemic metastasis of carcinoma

These case studies suggest that targeted therapies, such as bevacizumab and imatinib, may offer promising therapeutic options for patients with PTTM. While further research is needed to establish definitive treatment guidelines, these studies provide valuable insights into the potential benefits of these agents in managing PTTM-related complications. It's important to note that the referred studies are case reports. While valuable for generating hypotheses and describing rare occurrences, case reports have inherent limitations that should be considered. These limitations include limited generalizability, lack of a control group, potential for bias, limited statistical power, and a retrospective nature. Further research, such as larger observational studies or randomized controlled trials, is often needed to confirm the findings and draw more definitive conclusions.

Future research should focus on identifying additional biomarkers that can predict patient response to these therapies and developing novel therapeutic strategies that target the underlying pathophysiological mechanisms of PTTM. These mechanisms may include the activation of coagulation cascade and release of inflammatory mediators, fibrocellular subintimal proliferation, and smooth muscle cell colonization [28]. Several potential biomarkers have been associated with PTTM, including VEGF, PDGF, osteopontin, and TF [35]. These biomarkers are involved in pathways that contribute to the pathophysiology of PTTM, including angiogenesis, coagulation, and cellular proliferation. Further research is needed to validate their utility in clinical practice and to explore their potential in early diagnosis and targeted therapy for PTTM.

## Conclusions

In conclusion, the association between adenocarcinoma and PTTM presents a significant clinical challenge. Current treatment strategies for PTTM in adenocarcinoma patients primarily focus on managing the underlying malignancy and addressing thrombotic complications. Anti-angiogenic therapy bevacizumab and a PDGF receptor antagonist imatinib have shown promising results in some cases.

Future research should focus on the early detection of PTTM in adenocarcinoma patients, understanding the molecular mechanisms underlying the association between these two diseases, and developing innovative therapeutic approaches that target the aforementioned specific pathophysiological processes involved. By advancing our understanding of PTTM in adenocarcinoma, we can improve patient outcomes and ultimately save lives.
